# Bumetanide, an Inhibitor of NKCC1 (Na-K-2Cl Cotransporter Isoform 1), Enhances Propofol-Induced Loss of Righting Reflex but Not Its Immobilizing Actions in Neonatal Rats

**DOI:** 10.1371/journal.pone.0164125

**Published:** 2016-10-26

**Authors:** Yukihide Koyama, Tomio Andoh, Yoshinori Kamiya, Tomoyuki Miyazaki, Koichi Maruyama, Takayuki Kariya, Takahisa Goto

**Affiliations:** 1 Department of Anesthesiology and Critical Care Medicine, Yokohama City University Graduate School of Medicine, Yokohama, Japan; 2 Department of Anesthesiology, Mizonokuchi Hospital, Teikyo University School of Medicine, Kawasaki, Japan; 3 Department of Anesthesiology, Niigata University Graduate School of Medicine, Niigata, Japan; McLean Hospital/ Harvard Medical School, UNITED STATES

## Abstract

Gamma-aminobutyric acid (GABA) has been shown to induce excitation on immature neurons due to increased expression of Na+-K+-2Cl- co-transporter isoform 1 (NKCC1), and the transition of GABAergic signaling from excitatory to inhibitory occurs before birth in the rat spinal cord and spreads rostrally according to the developmental changes in cation-chloride co-transporter expression. We previously showed that midazolam activates the hippocampal CA3 area and induces less sedation in neonatal rats compared with adolescent rats in an NKCC1-dependent manner. In the present study, we tested the hypothesis that propofol-induced loss of righting reflex (LORR) but not immobilizing actions are modulated by NKCC1-dependent mechanisms and reduced in neonatal rats compared with adolescent rats. We estimated neuronal activity in the cortex, hippocampus and thalamus after propofol administration with or without bumetanide, an NKCC1 inhibitor, by immunostaining of phosphorylated cyclic adenosine monophosphate-response element binding protein (pCREB). We studied effects of bumetanide on propofol-induced LORR and immobilizing actions in postnatal day 7 and 28 (P7 and P28) rats. The pCREB expression in the cortex (P = 0.001) and hippocampus (P = 0.01) was significantly greater in the rats receiving propofol only than in the rats receiving propofol plus bumetanide at P 7. Propofol-induced LORR or immobilizing effects did not differ significantly between P7 and P28. Bumetanide significantly enhanced propofol-induced LORR (P = 0.031) but not immobilization in P7 rats. These results are partially consistent with our hypothesis. They suggest that propofol may activate the rostral but not caudal central nervous system dependently on NKCC1, and these differential actions may underlie the different properties of sedative and immobilizing actions observed in neonatal rats.

## Introduction

It has been shown that γ-aminobutyric acid (GABA) exerts excitatory actions on immature neurons due to the increased expression of Na+-K+-2Cl- co-transporter isoform 1 (NKCC1) [1, 2]. In immature rat neurons, expression of NKCC1 is dominant compared with that of K+-Cl- co-transporter isoform 2 (KCC2), and this balance is reversed during early brain development due to the strong upregulation of KCC2 [1, 3]. NKCC1 works to uptake Cl-1 into the cytoplasm, whereas KCC2 primarily acts to remove Cl-1 from the cytoplasm. Therefore, the equilibrium potential for Cl-1 (ECl) becomes more positive than the resting membrane potential [1–3]. As a result, the activation of GABA_A_ receptors induces Cl-1 efflux and neuronal depolarization in certain regions of the neonatal rat brain such as the hippocampus and cortex [4, 5].

In our previous study, we found that midazolam, a GABA_A_ receptor-stimulating sedative agent, exhibited reduced sedative actions in neonatal rats and that bumetanide, an inhibitor of NKCC [1, 2, 6] enhanced the sedative effects of midazolam in neonatal but not in adolescent rats [7]. Estimation of neuronal activity using expression of phosphorylated cyclic adenosine monophosphate-response element-binding protein (pCREB) revealed that midazolam activated the hippocampal CA3 region but not the thalamus in neonatal rats, and the increased activity was inhibited by bumetanide [7]. The regional difference in midazolam-induced changes in neural activity seems to be explained by the findings that the transition of GABA signaling from excitation to inhibition occurs earlier in the caudal part of the central nervous system (CNS) than in the rostral part [3, 8]. This difference is due to regional differences in the timing of the developmental change in the balance of NKCC1 and KCC2 expression [8, 9]. It is also known that the spinal cord undergoes the transition of GABA signaling before birth in rodents [8].

There are two major components in the actions of general anesthetics: sedative actions inducing a decreased level of arousal and immobilizing actions inducing a lack of motor responses to noxious stimuli [10]. Propofol, a widely used intravenous anesthetic, induces sedative effects mainly through enhancing GABA actions on GABA_A_ receptors similar to midazolam. However, unlike midazolam, it has other targets of action, such as inhibition of voltage-dependent Na channels and HCN1 channels [11, 12]. Although propofol exhibits only minor analgesic potency clinically, the immobilizing effect of propofol is known to mainly be mediated by actions on the spinal cord and involves GABA_A_ receptors [13, 14]. We aimed to test our hypothesis that the sedative but not immobilizing actions of propofol would be reduced in immature rats compared with mature ones and that its sedative but not immobilizing actions would be modulated by bumetanide in immature rats. We tested this hypothesis using neonatal (7-day old) and adolescent (28-day old) rats. We assessed the sedative effect of a moderate dose of propofol with or without bumetanide by the loss of righting reflex (LORR), and we also studied the immobilizing effect of a high dose of propofol with or without bumetanide by the loss of tail-pinch withdrawal response (LTPWR). In addition, neuronal activities of the cortex, hippocampus and the thalamus were estimated by immunostaining of pCREB-positive cells. Our results are partially consistent with our hypothesis and add new insight into the neuropharmacology of GABA_A_ receptor-stimulating anesthetic agents in the immature CNS.

## Materials and Methods

All animal procedures and protocols used in this study were approved by the Yokohama City University Institutional Animal Care and Use Committee (Yokohama, Japan). The experimental protocol number issued by our institution was F-A-12-032. Sprague-Dawley rats at the ages of 7 and 28 days were used in this study. We obtained all animals from Japan SLC Corporation (Shizuoka, Japan). All animals were kept in cages with their littermates and mothers in a temperature-controlled animal care facility room with a 12-h light/dark cycle at our institution. We made all efforts to minimize animal suffering and the number of animal used. All drugs were given by intraperitoneal injection.

### Behavioral Assays

LORR was used for assessments of the levels of sedation in this study. We randomly divided 16 rats into equal-sized groups according to the combination of the drugs to be administered, resulting in two groups of 8 rats each for postnatal day 7 (P7) and day 28 (P28) rats. The two drug combinations were (1) propofol (Maruishi Pharmaceuticals Co, Ltd., Osaka, Japan) 50 mg/kg 1 h after bumetanide 3.6 mg/kg (bumetanide + propofol group) and (2) propofol 50 mg/kg 1 h after saline at the same volume as the vehicle for bumetanide (saline + propofol group). After administration of the drugs, each animal was placed in the cage holding its mother and littermates. The duration of LORR was measured on a flat stage 30 min after administration of the second drug by recording with a stopwatch the time required for the rat to turn over to the upright position after placing it in the supine position. Three measurements were made, and the mean duration was calculated. If the rats failed to right themselves within 60 s, we recorded the duration to be 60 s and turned them to the upright position and continued the measurements.

LTPWR was used as a surrogate measure for immobilization [15, 16]. In this assay, we also randomly divided 16 rats into two groups of 8 rats each according to the two combinations of the drugs to be administered for P7 and P28 rats, respectively. The two drug combinations were (1) propofol 75 mg/kg 1 h after bumetanide 3.6 mg/kg) (bumetanide + propofol group) and (2) propofol 75 mg/kg 1 h after saline at the same volume as the vehicle for bumetanide (saline + propofol group). Immediately after administration of the second drug, rats were placed in a translucent plastic chamber (30×43×14 cm) in a thermostatic bath (36±2°C). The chamber was continuously flushed with 100% oxygen at an approximate rate of 6.0 l/min. A bulldog-type vessel clamp (45 mm in size; Natsume Seisakusho Co., Ltd., Tokyo, Japan) was placed at the base of an animal’s tail for 3 s at 1-min intervals from a time point of 15 min after administration of the second drug in this chamber. The lid of the chamber was removed only during the measurement of tail withdrawal response.

In these experiments, we used a 50-mg/kg dose of propofol for the assessment of its sedative effects because this dose induced sufficient sedation in P28 rats, and neither P7 nor P28 rats exhibited visible signs of cyanosis on room air throughout the experiment. However, in the pilot experiments, most of the rats that received 50 mg/kg propofol responded to the tail pinch test, and 100 mg/kg propofol was often lethal for P7 rats. A previous report showed that a single intraperitoneal injection of 75 mg/kg propofol prevented neonatal rats from responding to the tail pinch test [17]. Therefore, we used this dose for the assessment of the immobilizing actions of propofol. Because this dose of propofol induced cyanosis in some of the neonatal rats on room air, we applied oxygen after administration of 75 mg/kg propofol. No rats exhibited visible cyanosis under oxygen administration. Bumetanide was dissolved in dimethyl sulfoxide (DMSO) at the concentration of 50 mg/ml, and then this solution was diluted 100 times with saline that was alkalinized by adding 100 μl of 1N NaOH to 100 ml of saline. The saline administered in the saline group contained the same volume of DMSO and 1N NaOH.

### Immunohistochemistry

Twelve rats were randomly divided into equal-sized groups according to the three combinations of the drugs to be administered, resulting in 3 groups of 4 rats each for P7 and P28 rats, respectively. The three drug combinations were (1) propofol 50 mg/kg 1 h after bumetanide 3.6 mg/kg (bumetanide + propofol group), (2) propofol 50 mg/kg 1 h after saline at the same volume as the vehicle for bumetanide (saline + propofol group), and (3) Intralipos Injection 20%^™^ (Otsuka Pharmaceutical Co., Ltd., Tokyo, Japan) at half the volume of the 50 mg/kg propofol injection 1 h after saline at the same volume as the vehicle for bumetanide (control group). At 45 min after the second drug was administered, rats were deeply anesthetized by inhalation of isoflurane for approximately 10 s and perfused transcardially with 4% paraformaldehyde in 0.05 M phosphate-buffered saline before decapitation. Preparation of brain slices after decapitation and immunostaining of pCREB were performed by the methods previously described [7]. We selected two slices from each brain that were comparable with figures 37 and 39 in the atlas of Paxinos and Watson [18]. We selected the section of figure 39 for the cortex (retrosplenial granular cortex and retrosplenial granular b cortex) and hippocampal CA3 area, and the section of figure 37 for the thalamus to count the number of pCREB-positive cells in each area in the bilateral hemisphere of all samples. The images were photographed, and the pCREB-positive cells were counted with a Biorevo BZ 9000 microscope (Keyence Corporation, Osaka, Japan) by an operator blinded to the drug administrations. Because bumetanide alone had no influence on LORR compared with the control group in P7 or P28 rats in our previous experiment [7], we did not conduct behavior tests or pCREB immunohistochemistry after administration of bumetanide and saline in the current study.

### Statistics

Data are given as the mean with SD unless otherwise stated. For comparison of the durations of LORR or LTPWR, we used the Mann-Whitney U test to compare the durations between the two groups receiving different treatment within each age and those between P7 and P28 rats receiving the same treatment. Comparison of the number of pCREB-positive cells between the three groups within each age were analyzed by one-way ANOVA followed by Tukey post hoc tests. We used two-tailed tests in all comparisons. A P value of < 0.05 was considered statistically significant. Statistical analysis was performed with SPSS 17.0 for Windows (SPSS Inc., Chicago, IL).

## Results

The duration of LORR was significantly longer in the bumetanide + propofol group than in the saline + propofol group at P7 (P = 0.031, Table 1). However, there was no difference in the duration of LORR between the two different treatment groups at P28 (P = 0.96, Table 1). In the saline + propofol groups, the duration of LORR was shorter at P7 than that at P28. However, the difference between the two age groups did not reach statistical significance (P = 0.057). In contrast to the results of LORR, there was no significant difference in the duration of LTPWR between the bumetanide + propofol group and the saline + propofol group in either P7 or P28 rats (Table 2). The durations of LTPWR did not differ significantly in the saline + propofol group between the two age groups (P = 0.38).

**Table 1:** Durations of Loss of Righting Reflex in the Saline + Propofol and Bumetanide + Propofol Groups in P7 and P28 Rats.

	Saline + Propofol (s)	Bumetanide + Propofol (s)	P value
P7 (n = 8)	21.4 (9.9, 37.1) [2.0–60]	53.7 (45.7, 60) [43.6–60]	0.031
P28 (n = 8)	60 (59.1, 60) [5.2–60]	60 (55.6, 60) [27.1–60]	0.96

P7: postnatal day 7, P28: postnatal day 28. Values are expressed as median, (25^th^, 75^th^ percentile) and [range].

**Table 2:** Durations of Loss of Tail-pinch Withdrawal Reflex in the Saline + Propofol and Bumetanide + Propofol Groups in P7 and P28 Rats.

	Saline + Propofol (min)	Bumetanide + Propofol (min)	P value
P7 (n = 8)	1.5 (0.75, 4) [0–5]	3 (0.75, 4.25) [0–7]	0.59
P28 (n = 8)	5 (2.25, 6.25) [0–16]	5.5 (3, 8) [0–12]	0.71

P7: postnatal day 7, P28: postnatal day 28. Values are expressed as median, (25^th^, 75^th^ percentile) and [range].

**Fig 1:**
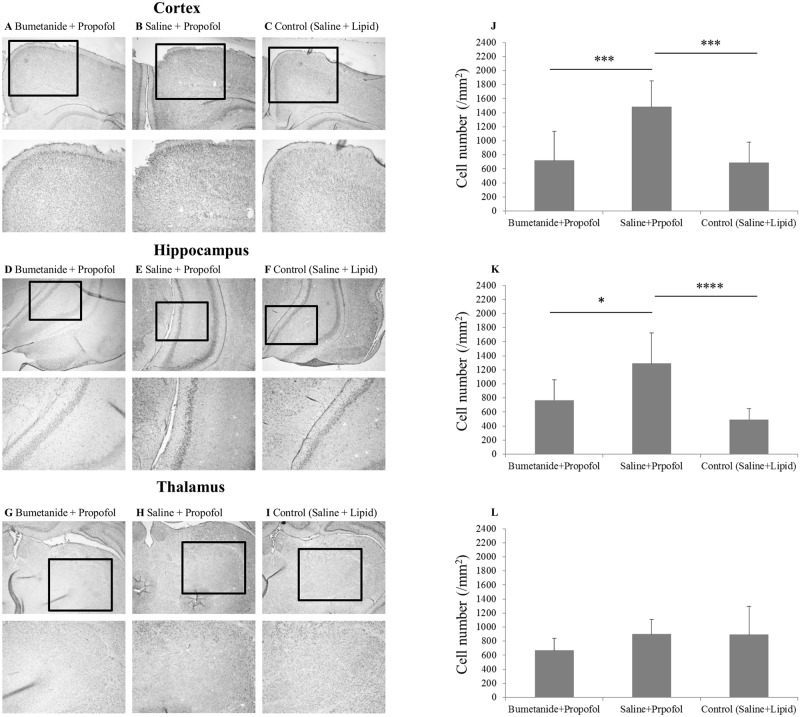
Changes in the expression of phosphorylated cyclic adenosine monophosphate-response element-binding protein (pCREB) after intraperitoneal administration of propofol in postnatal day 7 rats. A, B and C (upper panels: ×4 magnification; lower panels: ×8 magnification) are photomicrographs of pCREB immunostaining in the cortex (retrosplenial granular cortex and retrosplenial granular b cortex) of rats. D, E and F (upper panels: ×4 magnification; lower panels: ×8 magnification) are those of pCREB immunostaining in the hippocampal CA3 area. G, H and I (upper panels: ×4 magnification; lower panels: ×8 magnification) are those of pCREB immunostaining in the thalamus. Graphs in J, K and L show the number of pCREB-positive cells in the cortex, hippocampus and thalamus, respectively. Data are given as mean and SD. *P = 0.01, ***P = 0.001 and ****P<0.001, respectively. Data are derived from 4 slices in each group.

**Fig 2:**
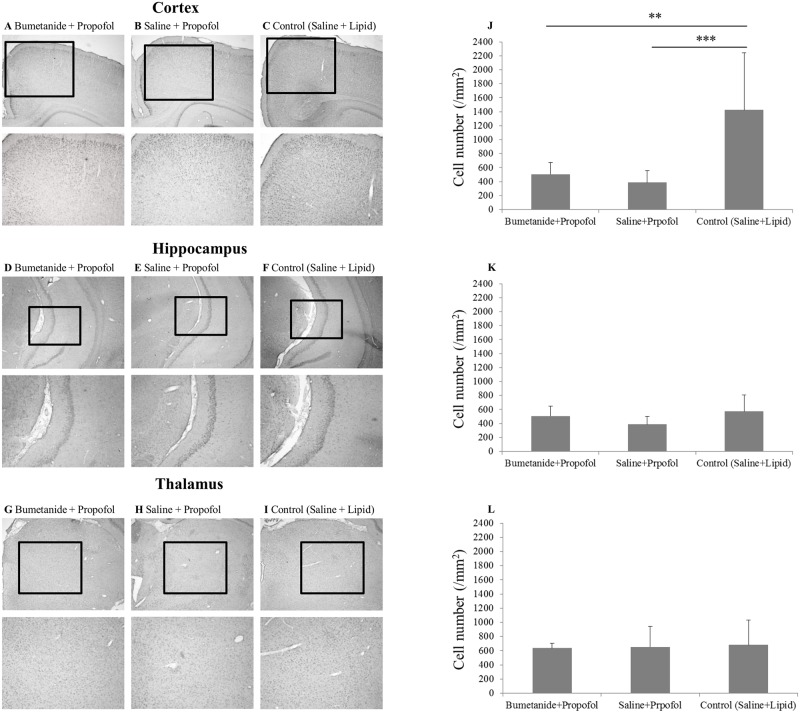
Changes in the expression of phosphorylated cyclic adenosine monophosphate-response element-binding protein (pCREB) after intraperitoneal administration of propofol in postnatal day 28 rats. A, B and C (upper panels: ×4 magnification; lower panels: ×8 magnification) are photomicrographs of pCREB immunostaining in the cortex (retrosplenial granular cortex and retrosplenial granular b cortex) of rats. D, E and F (upper panels: ×4 magnification; lower panels: ×8 magnification) are those of pCREB immunostaining in the hippocampal CA3 area. G, H and I (upper panels: ×4 magnification; lower panels: ×8 magnification) are those of pCREB immunostaining in the thalamus. Graphs in J, K and L show the number of pCREB-positive cells in the cortex, hippocampus and thalamus, respectively. Data are given as mean and SD. **P<0.01 and ***P = 0.001, respectively. Data are derived from 4 slices in each group.

The number of pCREB-positive cells in the neocortex (retrosplenial granular cortex and retrosplenial granular b cortex) was significantly higher in the saline + propofol group than in the bumetanide + propofol (P = 0.001) and control groups (P = 0.001) of P7 rats (Fig 1J; 1480.1±371.2, 715.8±416.8, and 685.3±295.4/mm^2^ for the saline + propofol, bumetanide + propofol, and control groups, respectively). Also the number of pCREB-positive cells in the hippocampal CA3 area was significantly higher in the saline + propofol group (1289.3±434.7/mm^2)^ than in the bumetanide + propofol (766.1±291.9/mm^2^) (P = 0.01) and control groups (490.9±153.6/mm^2^) (P<0.001) of P7 rats, respectively (Fig 1K). However, there were no significant differences in the number of pCREB-positive cells in the thalamus between the three groups at P7 (Fig 1L). Similarly, no significant differences were found in the thalamus between the three groups at P28 (Fig 2L). In contrast to the results of the neonates, however, there were no significant differences in pCREB expression in the hippocampal CA3 area between the three groups in P28 rats (Fig 2K). Interestingly, the number of pCREB-positive cells in the neocortex was significantly lower in the saline + propofol (386.3±173.4/mm^2^) (P = 0.001) and the bumetanide + propofol groups (504.1±170.5/mm^2^) (P<0.01) than in the control group (1426.0±815.7/mm^2^) in P28 rats, respectively (Fig 2J). In contrast to P7 rats, bumetanide pretreatment induced no significant changes in pCREB expression compared with the saline-pretreated propofol group in all three regions in P28 rats.

## Discussion

In neonatal rats, we found that propofol increased pCREB expression in the cortex and hippocampus CA3 regions but not in the thalamus and that bumetanide pretreatment inhibited the increase in pCREB expression in the cortex and hippocampus but did not affect the expression in the thalamus. We also found that propofol-induced LORR tended to be reduced in neonatal rats compared to that in adolescent rats, but the age-dependent difference just failed to reach statistical significance. The immobilizing actions of propofol were comparable between the two ages, and bumetanide pretreatment enhanced propofol-induced LORR but not the immobilizing actions in P7 rats. These results are partially consistent with our hypothesis and suggest that the differential activation of the caudal and rostral CNS by propofol may depend on NKCC1 and may underlie the difference in the properties of sedative and immobilizing actions observed in neonatal rats.

In our previous study, we found that midazolam induced significantly less sedation in a bumetanide-sensitive manner in P7 rats compared with that in P28 rats [7], and we hypothesized that the sedative actions of propofol would have exhibited the same age-dependent difference. However, the age-dependent difference for propofol did not reach statistical significance (P = 0.057). The possible reasons for this might include the small sample size or the mechanistic difference in sedative actions of midazolam and propofol. Propofol-induced sedation may involve mechanisms other than GABA_A_ receptor stimulation [11, 12], whereas midazolam has minimal effects on other neural targets. We found that pretreatment with bumetanide significantly enhanced propofol-induced LORR in P7 rats but had no effect in P28 rats, in a similar manner to that of midazolam. These results suggest that the sedative actions of midazolam and propofol are qualitatively similar in terms of the developmental change in NKCC1 dependency [7].

The results from the tail-pinch study showed that the propofol-induced immobilizing effects in neonatal rats were comparable to those in adolescent rats and were not modulated by bumetanide, in accordance with our hypothesis. Although we have not studied the immobilizing actions of midazolam, an earlier study indicated that midazolam decreased the hind limb withdrawal threshold to mechanical noxious stimuli in the P3 mouse compared with that in adolescent and adult animals [19]. The direct comparison of the results of these studies is difficult because of differences in the age, parameters, and methods used. However, it may be noteworthy that the midazolam-induced immobilizing effect is suggested to be mediated through supraspinal actions [20] and that the decreased withdrawal threshold induced by midazolam at P3 was not observed when administered intrathecally [19]. Unlike midazolam, the propofol-induced immobilization is considered to depend on spinal actions [13, 20]. Therefore, it is suggested that the differences in the sites of actions responsible for immobilizing effects may contribute to different properties of the immobilizing effects of midazolam and propofol observed in immature animals.

pCREB is one of the activity-dependent transcription regulators and has been considered one of the major markers of Ca^2+^ influx and neuronal excitation [21, 22]. In this study, we found that propofol induced significant up-regulation of pCREB in the cortex and the hippocampal CA3 area in P7 but not P28 rats and that bumetanide inhibited these changes. These results suggest that the activation of GABA_A_ receptors increases activity in the developing rat endbrain in an NKCC1-dependent manner. The cortex and subcortical areas have key roles in the sedative response to GABAergic general anesthetics through an endogenous sleep pathway [23]. Furthermore, Ma et al. showed that the hippocampus participates in the sedative actions of general anesthetics [24]. Therefore, our results suggest that propofol-induced activation of the developing endbrain may hamper sedative actions in neonatal rats.

We used the righting reflex to estimate sedation because LORR has been successfully used as a surrogate measure for estimating sedative actions of anesthetics in rodents for many years [25]. The righting reflex is basically governed from below the decerebration level, in the midbrain and more caudal CNS, in quadrupeds [26]. However, it is known that the higher brain structures including the cortex supplement the righting responses [26–28]. The brainstem arousal pathways and thalamocortical system are considered to be important targets for propofol-induced hypnosis [25, 29]. Although we did not observe down-regulation of pCREB in the thalamus, propofol likely inhibits some parts of the brainstem and thalamus in neonatal rats, leading to inhibition of the righting reflex and sedation [30], because GABA_A_ receptor signaling is inhibitory in these regions even at P7. We speculate that in the presence of bumetanide, propofol-induced LORR was enhanced because bumetanide may suppress the propofol-induced activation of the cortex without affecting inhibition of the caudal CNS in P7 rats. As for propofol-induced immobilization, it has been shown that this action was mediated by propofol’s action on the spinal cord and that decerebration did not affect the propofol requirement for inducing immobility to tail pinch stimuli [13]. These earlier findings may well explain our results that propofol-induced inhibition of TPWR was not affected by bumetanide even though bumetanide suppressed the propofol-induced activation of the cortex. Therefore, it seems that activity of the higher brain such as the cortex differentially modify propofol-induced LORR and immobilization.

In contrast to the results of the cortex and hippocampus of P7 rats, no activation was induced by propofol in the thalamus at P7. This result is also consistent with the results of our previous report [7] and those by Glykys et al. [9] They showed that the effects of the activation of GABA_A_ receptor are inhibitory in the ventroposterior thalamus but excitatory in the neocortex in neonatal rats due to the earlier maturation of the expression pattern of cation-chloride co-transporters (CCC) in this thalamic region [9]. It is known that maturation of the CCC expression pattern in the spinal cord and the brainstem occurs before birth in rodents and proceeds from the caudal to rostral parts of the CNS during development [8]. Because bumetanide failed to affect the action of propofol on pCREB expression in the thalamus at P7 in the present study, it would be likely that the transition of GABA_A_ receptor signaling has already occurred in the more caudal part including the spinal cord at P7. The lack of propofol-induced activation of the caudal CNS is consistent with the results of the tail-pinch test and the lack of modulation of immobility by bumetanide in neonatal and adolescent rats. Taken together, our results would suggest that these differential effects of bumetanide on sedative and immobilizing actions might be explained by the earlier maturation of the GABA_A_ receptor-mediating signaling in the caudal CNS being responsible for the immobilizing actions compared with that in the rostral brain due to the regional difference in the development of CCC expression within the CNS.

In regard to the in vivo actions of bumetanide, pharmacokinetic studies have indicated poor penetration of bumetanide into the blood brain barrier (BBB)-protected CNS even in neonatal rodents with immature BBB [31, 32]. These studies have questioned the notion that the effects of systemic administration of bumetanide are mediated by actions on NKCC1 in the BBB-protected CNS [32, 33]. However, a number of studies have provided evidence that intraperitoneal administration of bumetanide at doses lower than those in our study depresses hyperactivity of the cortex and hippocampus in immature rodents [6, 34–36], and these findings are consistent with the in vitro demonstration of NKCC1 inhibition of central neurons by bumetanide. Future studies are warranted to directly prove that systemic administration of bumetanide inhibits NKCC1 in immature central neurons and changes ECl leading to the modulation of GABA_A_ receptor signaling in vivo.

Our study has several limitations. First, we did not conduct either a Ca^2+^ imaging study for the measurement of the changes of [Ca^2+^]i or an electrophysiological study in the three regions. Therefore, we did not obtain direct evidence that the propofol-induced activation of GABA_A_ receptors is followed by depolarization due to NKCC1-dependent changes in ECl. Second, we did not examine pCREB expression in the spinal cord important for LTPWR induced by propofol. Therefore, our interpretation of the results of LTPWR is speculative and needs to be validated by further studies examining changes in pCREB expression of the spinal cord induced by propofol with or without bumetanide. Third, we did not obtain a dose-response relationship for either the sedative or the immobilizing actions of propofol and compare the effective doses for 50% response among the different conditions. However, it was impossible to study a wide range of propofol doses in neonatal rats because the safety margin of propofol administration was very small. For example, propofol at 100 mg/kg frequently resulted in severe cyanosis and death even under oxygen administration at P7. Fourth, we compared the actions of propofol in P7 and P28 rats at the same doses; however, these doses have not been proven to be equipotent for the studied ages. Fifth, the sample size was not calculated based on a pilot study. Therefore, we should consider the possibility of a Type 2 statistical error in the results. However, we previously demonstrated age- and bumetanide-dependent differences in the sedative effects of midazolam in 6 rats for each group [7]. In the current study, we used 8 rats for each group assuming that 8 rats in each group are sufficient for the behavior assays studied. Sixth, we related the effects of bumetanide on behavior studies to those on pCREB expression in our study. We used a higher dose of propofol for LTPWR than that for immunohistochemistry. It was preferable to relate the findings at the same dose of propofol. However, we mainly focused on the effects of bumetanide on propofol-induced changes in LTPWR and thalamic pCREB expression. It seems unlikely that propofol doses would cause drastic or qualitative changes in the effects of bumetanide on propofol-induced alteration of thalamic pCREB expression, considering the putative mechanisms of bumetanide’s actions.

Clinical implications of our results are limited because human data for developmental changes in ECl of central neurons are unavailable. The data on the developmental changes in NKCC1 and KCC2 expression in the human brain are inconsistent. An earlier report indicated that the human cortex undergoes similar developmental changes during the perinatal period [6], whereas recent systematic studies have shown that KCC2 upregulation starts early in the prenatal period and reaches a high level at birth [3, 37, 38]. However, GABAergic anti-convulsants are shown to be less effective in the inhibition of cortical seizure activity than in the inhibition of motor activity in neonatal seizure [39, 40], suggesting similar differential maturation of GABA_A_ receptor signaling between the rostral and caudal CNS in humans. Our results also raise the possibility that propofol-induced suppression of the higher brain may be reduced in human early neonates in spite of preserved depression of the lower CNS.

In summary, our results suggest that propofol may increase the activity of the developing hippocampal CA3 area and neocortex possibly leading to reduced sedative effects in neonatal rats in a bumetanide-sensitive manner. In contrast, the immobilizing actions of propofol were not reduced or modulated by bumetanide in neonatal rats. These different properties of propofol-induced LORR and immobilizing actions in neonatal rats may be related to the different timings of the transition of GABA_A_ receptor signaling from excitatory to inhibitory in the caudal and rostral CNS due to regional differences in the development of CCC expression. To our knowledge, there are no studies investigating the different properties of the hypnotic and immobilizing actions of propofol in immature animals with special reference to maturation of GABA_A_ receptor function. Therefore, our findings are novel and provide new insights as to how anesthetics act on the immature CNS.

## Supporting Information

S1 Table Original data of behavioral assays. (XLSX)

S2 Table Original data of pCREB positive cells. (XLSX)
